# Interaction among *CYP2C8*, *GPIIIa* and *P2Y12* variants increase susceptibility to ischemic stroke in Chinese population

**DOI:** 10.18632/oncotarget.19991

**Published:** 2017-08-07

**Authors:** Xingyang Yi, Jing Lin, Yanfen Wang, Ju Zhou, Qiang Zhou

**Affiliations:** ^1^ Department of Neurology, People’s Hospital of Deyang City, Deyang 618000, Sichuan, China; ^2^ Department of Neurology, The Third Affiliated Hospital of Wenzhou Medical University, Wenzhou 325200, Zhejiang, China

**Keywords:** ischemic stroke, genetic polymorphism, cytochrome P450, platelet membrane receptor, glycoprotein IIIa

## Abstract

**Purpose:**

Genetic variants in cytochrome P450 (*CYP*)*,* platelet membrane receptor (*P2Y12, P2Y1),* and glycoprotein IIIa (*GPIIIa*) genes are associated with the efficacy of clopidogrel and adverse clinical events on ischemic stroke (IS) patients. However, few studies have assessed whether gene-gene interactions among these genes influence the risk of IS. The aim of the present study was to investigate the association of fifteen variants with IS and to determine whether these gene-gene interactions increase the risk of IS.

**Methods:**

Fifteen variants in *CYP3A4, CYP3A5, CYP2C8, CYP2C9, CYP2C19, P2Y12*, *P2Y1* and *GPIIIa* genes were examined using mass spectrometry methods in 396 patients with IS and 378 controls. Gene-gene interactions were analyzed using generalized multifactor dimensionality reduction (GMDR) methods.

**Results:**

Single-gene variant analysis showed no significant differences in the genotype distributions of the fifteen variants between IS patients and controls using the single-locus analytical approach. However, GMDR analysis showed a significant gene-gene interaction among rs17110453A>C, rs2317676A>G, and rs16863323C>T, which scored 10 for cross-validation consistency and 9 for the sign test (*P* = 0.016). Logistic regression analysis showed that high-risk interactions among rs17110453A>C, rs2317676A>G, and rs16863323C>T were independent risk factor for IS after adjusting for age, hypertension, diabetes mellitus, and hemoglobin A1C (*OR*=2.24, 95% *CI*: 1.17–5.62, *P*=0.005).

**Conclusions:**

The rs17110453A>C, rs2317676A>G, and rs16863323C>T three-loci interaction may confer a higher risk for IS. The combinatorial analysis used in this study may be helpful to elucidate complex genetic risk factors for IS.

## INTRODUCTION

Stroke has been considered a major worldwide health problem and is becoming one of the leading causes of mortality among the elderly [1]. It has been generally considered as a multifactorial and heterogeneous disorder caused by both conventional environmental risk factors and genetic factors [2]. Although the mechanisms remain unclear, genetic predisposition has been suggested to be a critical player in the pathogenesis of this disease [3].

Our previous studies have shown that genetic variants in cytochrome P450 (*CYP*), platelet membrane receptor (*P2Y12, P2Y1),* and glycoprotein IIIa (*GPIIIa*) genes are associated with the efficacy of clopidogrel and adverse clinical events on ischemic stroke (IS) patients in a Chinese population [4, 5]. However, it is unclear whether these genetic variants also play a role in pathogenesis of IS. The *CYP2* and *CYP3A* gene family encodes for the major epoxygenase enzymes, expressed predominantly in vascular endothelial cells and heart tissue, which metabolize Arachidonic acid (AA) into four epoxyeicosatrienoic acids (EETs) [6]. Our previous studies and one other study have shown that plasma CYP metabolite levels, including EETs are associated with IS [7-9]. Platelet activation plays a key role in the pathogenesis of IS [10]. Platelet membranes receptors (P2Y12, P2Y1) have been suggested to play a major role in the process of platelet aggregation, as well as arterial thrombosis [11, 12]. The fibrinogen receptor, which is a composite of 2 subunits, glycoprotein IIb (GPIIb) and glycoprotein IIIa (GPIIIa), is presumably the final common pathway of platelet activation, adhesion, and aggregation [13]. Growing evidence showed that single nucleotide polymorphisms (SNPs) in *P2Y12*, *P2Y1* and *GPIIb/IIIa* genes may influence responsiveness to antiplatelet medications [14, 15]. However, possible role of these genetic variants in relation to IS has received limited attention.

IS appears to be a disease that does not follow the Mendelian pattern of inheritance, suggesting that single-locus analysis may not be appropriate to investigate the genetic risk factors of IS [2]. Single-locus analysis may fail to detect significant variants that may exert significant influence on the pathogenesis of IS via synergistic interactions with other locus variants [16]. Thus, the search for gene variants linked to IS risk may be significantly enhanced by thoroughly investigating gene–gene interactions via alternative analytical methods, such as the generalized multifactor dimensionality reduction (GMDR) approach [17]. However, the relationship between these gene-gene interactions and IS risk has not been well addressed.

Despite some previous studies have shown that genetic variants in *CYP3A*, *CYP2, P2Y12*, *P2Y1* and *GPIIb/IIIa* genes are associated with the efficacy of clopidogrel and adverse clinical events on IS patients, currently there are few studies to investigate the association of these genetic variants and these gene-gene interactions with IS risk. We hypothesized that the interaction of these variants might confer a higher IS risk than a single variant in one gene. In this study, we examined the association of fifteen variants in *CYP3A4, CYP3A5, CYP2C8, CYP2C9, CYP2C19, P2Y12*, *P2Y1* and *GPIIIa* genes with the risks of IS. In addition, we investigated whether these gene-gene interactions increase the risk of IS in Chinese populations.

## RESULTS

### Clinical characteristics of the subjects

The baseline characteristics of the IS patients and controls are shown in Table 1. Compared with controls, the IS patients were older (*P*<0.001), had a higher prevalence of hypertension (*P*<0.001) and diabetes mellitus (*P*=0.032), and higher hemoglobin A1C levels (*P*<0.001). However, no statistically significant differences in the conventional risk factors, including smoking, alcohol intake, levels of low-density lipoprotein cholesterol (LDL-C), total plasma cholesterol (TC), triglycerides (TG), homocysteine, and body mass index, were identified between the two groups (*P* >0.05).

**Table 1:** Clinical characteristics

Characteristic	IS patients (n = 396)	Controls (n = 378)	*P* value
Age (years)	68.79 ± 11.11	64.98 ± 10.29	<0.001
Men (*n*, %)	235 (59.34)	222 (58.73)	0.924
Hypertension (*n*, %)	302 (76.26)	99 (26.19)	<0.001
Diabetes mellitus (*n*, %)	138 (34.85)	97 (25.66)	0.032
Body mass index (kg/m^2^)	24.10 ± 2.33	23.90 ± 2.62	0.221
Cigarette smoking (*n*, %)	165 (41.67)	159 (42.06)	0.942
Alcohol intake (*n*, %)	184 (46.46)	170 (44.97)	0.694
TG (mM)	1.96 ± 1.12	1.83 ± 1.02	0.182
TC (mM)	5.54 ± 1.36	5.36 ± 1.21	0.061
LDL-C (mM)	3.15 ± 1.27	2.99 ± 1.19	0.376
HDL-C (mM)	1.23 ± 0.38	1.26 ± 0.42	0.223
Homocysteine (mM)	12.73 ± 4.24	12.49 ± 4.36	0.462
Hemoglobin A1C (%)	6.62 ± 1.62	6.02 ± 1.64	<0.001
Previous treatment (n, %)			
Antihypertensive drugs	117 (29.5)	89 (23.5)	0.071
Hypoglycemic drugs	113 (28.5)	85 (22.5)	0.059
Statins	48 (12.1)	44(11.6)	0.912
Aspirin	51 (12.9)	45 (11.9)	0.823

IS, ischemic stroke; TG, triglycerides; TC, total cholesterol; LDL-C, low-density lipoprotein cholesterol; HDL-C, high-density lipoprotein cholesterol.

### Comparison of the genotype distributions between IS patients and controls

The genotype distributions of the fifteen variants examined in this study were consistent with the Hardy-Weinberg Equilibrium model (*P* >0.05). There were no significant differences in the frequencies of the genotypes of the fifteen variants between the IS patients and the controls (*P* >0.05, Table 2). Moreover, there were no significant difference in genotype frequencies between atherothrombotic (AT) and small artery disease (SAD) patients (*P >*0.05).

**Table 2:** Genotype comparison between the two groups (*n*, %)

	Patients (n = 396)	Controls (n = 378)	*P* value
*CYP 2C8* (rs17110453)			
AA	180 (45.45)	180 (47.62)	0.796
AC+CC	216 (54.55)	198 (52.38)	
*CYP 2C8*(rs1934980)			
CC	57 (14.39)	55 (14.55)	0.892
CT+TT	339 (85.61)	323 (85.45)	
CYP2C9*2 (rs1799853)			
CC	396 (100)	378 (100)	-
CYP2C9*3(rs1057910)			
AA	360 (90.91)	349 (92.33)	0.824
AC+CC	36 (9.09)	29 (7.67)	
*CYP3A5*(rs776746)			
AA	45 (11.36)	40 (10.58)	0.682
AG+GG	351 (88.64)	338 (89.41)	
CYP2C19*2 (rs4244285)			
GG	198(50.0)	182(48.1)	0.836
AG+AA	198(50.0)	196(51.9)	
CYP2C19*3 (rs4986893)			
GG	361(91.2)	355(93.9)	0.468
AG	35(8.8)	23(6.1)	
*P2Y1* (rs701265)			
AA	213(53.8)	219(57.9)	0. 325
AG+GG	183(46.2)	159(42.1)	
*P2Y1* (rs1371097)			
CC	204(51.5)	221(58.5)	0.206
TT+CT	192(48.5)	157(41.5)	
*P2Y1* (rs1439010)			
AA	206(52.0)	219(57.9)	0.213
AG+GG	190(48.0)	159(42.1)	
*P2Y12*(rs16863323)			
CC	88 (22.2)	95 (25.1)	0.522
TT+CT	308(77.8)	283 (74.9)	
*P2Y12* (rs9859538)			
GG	304(76.8)	280 (74.1)	0.563
AG+AA	92 (23.2)	98 (25.9)	
*GPIIIa* (rs2317676)			
AA	233 (58.8)	242 (64.0)	0.284
AG+GG	163(41.2)	136 (36.0)	
*GPIIIa* (rs11871251)			
AA	141(35.6)	121(32.0)	0.431
AG+GG	255(64.4)	257(68.0)	
*CYP3A4*(rs2242480)			
CC	214(54.0)	200(52.9)	0.942
TT+CT	182(46.0)	178(47.1)	

### Gene–gene interactions in the IS patients and controls

We then investigated the association of the high-order interactions of SNPs with IS using the GMDR method. There were significant gene-gene interactions. The best model for IS was rs17110453A>C, rs2317676A>G, and rs16863323C>T after adjusting the covariates, which scored 10 out of 10 for cross-validation consistency and 9 out of 10 for the sign test (*P*= 0.016, Table 3). The one-locus model was also computed for each variant. The prediction accuracies of these one-locus models by GMDR were 0.523, 0.614 and 0.546 (for rs17110453A>C, rs2317676A>G, and rs16863323C>T, respectively), yielding a minimum *P* value of 0.923. The significance of this interaction was further confirmed by a permutation test (*P* = 0.014), suggesting that the three genetic variants together significantly contributed to IS was due to the synergistic action of the three genes and not due to variation in one locus alone.

**Table 3:** Comparison of the best models, prediction accuracies, cross-validation consistencies, and *P* values identified by generalized multifactor dimensionality reduction analysis

Best model*	Training balanced accuracy	Testing balanced accuracy	Cross-validation consistency	Sign test (*P* value)
15	0.513	0.523	8/10	7 (0.179)
1,2	0.524	0.519	9/10	8 (0.324)
1, 2, 3	0.572	0.543	10/10	9 (0.016)
1, 2, 3, 4	0.568	0.604	7/10	7 (0.514)
1, 2, 3, 4, 5	0.612	0.518	10/10	8 (0.642)
1, 2, 3, 4, 5, 6	0.587	0.476	8/10	6 (0.687)
1, 2, 3, 4, 5, 6, 7	0.626	0.524	7/10	4 (0.817)
1, 2, 3, 4, 5, 6, 7, 8	0.641	0.524	8/10	5 (0.347)
1, 2, 3, 4, 5, 6, 7, 8, 9	0.662	0.528	5/10	6 (0.832)
1, 2, 3, 4, 5, 6, 7, 8, 9, 10	0.586	0.554	7/10	5 (0.644)
1, 2, 3, 4, 5, 6, 7, 8, 9, 10,11	0.519	0.476	6/10	6(0.368)
1, 2, 3, 4, 5, 6, 7, 8, 9, 10,11,12	0.474	0.513	4/10	5 (0.724)
1, 2,3, 4, 5, 6, 7, 8, 9, 10,11,12,13	0.638	0.543	8/10	4(0.375)
1, 2, 3, 4, 5, 6, 7, 8, 9, 10,11,12,13,14	0.576	0.617	7/10	7 (0.247)
1, 2, 3, 4, 5, 6, 7, 8, 9, 10,11,12,13,14,15	0.552	0.514	8/10	5 (0.267)

* Numbers 1-15 represent rs17110453, rs2317676, rs16863323, rs4244285, rs11871251, rs776746, rs1371097, rs701265, rs1439010, rs2242480, rs9859538, rs4986893, rs1934980, rs1799853, and rs1057910, respectively.

Table 4 shows associations between IS and the different combinations of genotypes compared with wild-type genotypes rs17110453AA, rs2317676AA, and rs16863323CC. The three interactions making large contributions to this model were among rs17110453CC, rs2317676GG, and rs16863323TT; rs17110453CC, rs2317676GG/AG, and rs16863323TT; rs17110453CC, rs2317676AG, and rs16863323CT. The estimated risk of IS was significantly higher in individuals with rs17110453CC, rs2317676GG, and rs16863323TT as compared with patients harboring rs17110453AA, rs2317676AA, and rs16863323CC (odds ratio [OR] = 2.46, 95% CI: 1.18-5.96, *P* = 0.004). These results indicate that the interaction of multiple genes conferred a higher risk for stroke than did any single variant alone.

**Table 4:** Associations between cerebral infarction and genotype combinations

rs17110453	AA	CC	CC	CC	AC	CC, AC	CC	CC, AC
rs16863323	CC	TT	TT	CT	CT	TT	TT, CT	TT, CT
rs2317676	AA	GG	AG, GG	AG	AG	GG	GG	GG, AG
OR	1 *	2.46	1.97	2.23	1.07	1.14	1.08	1.12
95% CI	-	1.18-5.96	1.02-3.95	1.08-4.17	0.85-2.07	0.71-1.85	0.66-2.14	0.72-1.94
*P* value	-	0.004	0.037	0.021	0.317	0.284	0.476	0.603

*Non-risk genotype for each genetic factor was used as the reference OR. OR, odds ratios; CI, confidence interval.

For a prior probability of 0.1, assuming that the OR for specific genotype was 0.67/1.50 (protection/risk), with statistical power of 0.986, the false-positive report probability (FPRP) values were 0.173 for an association of high-risk interactive genotypes among rs17110453A>C, rs2317676A>G, and rs16863323C>T, with an increased risk of IS in all individuals. Because the probability to be a false-positive result was <20%, positive association of interaction among rs17110453A>C, rs2317676A>G, and rs16863323C>T three-loci with IS risk were considered noteworthy findings.

### Logistic regression analysis of risk factors for IS

The relative risk conferred by the combinations of variants in the three genes was considered as an interactive variable. The combinations of rs17110453CC, rs2317676GG, and rs16863323TT; rs17110453CC, rs2317676GG/AG, and rs16863323TT; rs17110453CC, rs2317676AG, and rs16863323CT were considered as high-risk interactive variable, with assigned as one; and other combinations of rs17110453A>C, rs2317676A>G, and rs16863323C>T as low-risk interactive variable, with assigned as zero. Logistic regression analysis showed that the high-risk interactive genotypes among variants in rs17110453A>C, rs2317676A>G, and rs16863323C>T predicted a significantly higher risk of IS, including adjustments for age, hypertension, diabetes mellitus, or hemoglobin A1C (OR = 2.24, 95% CI: 1.17–5.62, *P* = 0.005; Table 5).

**Table 5:** Multiple regression analysis of the major risk factors for IS

	Wald	OR	95% CI	*P* value
Age	5.23	1.24	1.08–2.36	0.014
Hypertension	14.23	5.22	2.4–11.8	< 0.001
Diabetes mellitus	0.85	0.82	0.72–1.36	0.128
High-risk interactive genotypes	8.8	2.24	1.17–5.62	0.005
Hemoglobin A1C	0.92	0.94	0.86–1.77	0.243

IS, ischemic stroke; OR, odds ratio; CI, confidence interval.

### Effect of high-risk interactive genotypes on platelet aggregation activity in IS patients

There were no significant differences in the activity of platelet aggregation among the 15 variants on admission. However, the platelet aggregation whether AA - induced or adenosine diphosphate (ADP) - induced was significantly higher in patients carrying high-risk interactive genotypes than in patients without carrying high-risk interactive genotypes on admission (Table 6).

**Table 6:** Effect of high-risk interactive genotypes on platelet aggregation activity in IS patients

	Platelet aggregation(%)	
	AA-induced	ADP-induced
High-risk interactive genotypes		
No (n=284)	87.3 ± 13.6	88.2 ± 14.2
Yes(n=112)	91.7 ± 10.6	92.3 ± 11.7
*P* value	<0.001	0.004

IS, ischemic stroke; AA, arachidonic acid; ADP, adenosine diphosphate.

## DISCUSSION

The possible role of genetic variation in *CYP, P2Y12*, *P2Y1* and *GPIIb/IIIa* genes in IS predisposition or prognosis has not yet been thoroughly investigated. The aim of this study was to examine potential associations of these variants with IS in a Chinese population. The present study identified no significant differences among the fifteen variants of *CYP, P2Y12*, *P2Y1* and *GPIIb/IIIa* genes between the IS patients and the healthy controls using the single-locus analytical approach. However, GMDR analysis revealed that rs17110453A>C, rs2317676A>G, and rs16863323C>T had a combinatorial effect to increase IS risk.

The *CYP2* and *CYP3A* gene family encodes for the major epoxygenase enzymes, the possible role of these variations in the development and prognosis of stroke remains unclear. Suh *et al.*[18] revealed that *CYP3A5* non-expression genotype (CYP3A5*3) was associated with an increased frequency of atherothrombotic events within six months after coronary angioplasty patients, whereas other studies did not confirm any association between *CYP3A5* genetic variants and the antiplatelet effect of clopidogrel [19]. Yi *et al.* [20] have reported that the genetic polymorphisms of *ALOX5AP* and *CYP3A5* increase susceptibility to ischemic stroke and are associated with atherothrombotic events in stroke patients. In Japanese population, genotypes for *CYP3A4* and *CYP3A5* were not associated with IS, but *CYP3A4* may be associated with intracerebral hemorrhage and subarachnoid hemorrhage [21]. Our previous studies showed that *CYP2C8* rs17110453 and *EPHX2* rs751141 two-locus interaction confered a significantly higher risk for IS [9, 22]. However, Marciante *et al.* [23] revealed that there was no association between variation in *CYP2C8* or *CYP2C9* and myocardial infarction or stroke.

Platelet membranes receptors and glycoprotein IIIa play a major role in the platelet activation and arterial thrombosis [12, 13]. Some studies found that *P2Y12* polymorphisms were associated with poorer vascular outcomes, and testing for these polymorphisms may be valuable in the identification of patients at risk for recurrent ischemic events [24]. However, Zee *et al.* [25] found no association of the *P2RY12* variants or the haplotype H2 with incident myocardial infarction (MI) or IS. A systematic review and meta-analysis has shown that carriage of the PlA2 polymorphism of *GPIIIa* is a risk factor for ischemic strokes, and specifically those of cardioembolic and large vessel origin [26]. In Mexican individuals, glycoprotein IIIa PIA1/A2 polymorphism represents a risk factor for myocardial infarction but not for idiopathic ischemic stroke [27]. Our present study also found no association between variants in *P2Y12, P2Y1, GPIIIa* genes and IS using the single-locus analytical approach.

There may be a number of potential causes for the contradiction results of these studies. The first reason may be attributed to the racial differences in the population of IS patients being investigated. A second explanation may be the complexity of IS etiology itself. As a matter of fact, it is highly likely that the pathogenesis of IS requires several variations, each with minor effects and potentially undetectable effects [25]. Therefore, a linkage analysis, which is used to investigate single-gene disorders, seems unsuitable for genetic studies on IS. Furthermore, other social differences also exist between these populations, which could potentially alter the environmental risk to which the patients are exposed.

The most noteworthy finding in the present study was made via a GMDR approach. Despite not being able to identify significance in any single locus variant, GMDR analysis revealed interesting synergistic effects of a gene variant–gene variant interaction. GMDR analysis revealed that rs17110453A>C, rs2317676A>G, and rs16863323C>T had a combinatorial effect to increase IS risk. Specifically, the risk for IS was noted to be increased by 2.24-fold in individuals carrying high-risk combination of genotypes of rs17110453A>C, rs2317676A>G, and rs16863323C>T, indicating that the three-loci interactions may plays a key role in the genetic predisposition to IS.

However, the nature of the interactions among the three gene variants is unclear. One possible explanation is that the three-factor interactions enhance the platelet activation, which plays a key role in the pathogenesis of IS [10]. The *CYP2* gene family encodes the major CYP epoxygenase enzymes. EETs exert vascular relaxation effects, inhibit platelet adhesion [28], and have diverse protective roles in the cardiovascular system [29]. The *CYP2C8* rs17110453 polymorphism decreases the activity of CYP2C8, thereby decreasing the levels of circulating EET metabolites, and thus increasing the risk for IS and IS-related injuries [9]. Platelet membranes receptors and glycoprotein IIIa play a major role in the platelet activation and arterial thrombosis. *P2Y12* rs16863323, *GPIIIa* rs2317676 encodes platelet membranes receptors and glycoprotein IIIa, respectively. Fontana *et al.* [30] has revealed ADP-induced platelet aggregation is associated with a haplotype of the *P2Y12* receptor gene. *GPIIIa* rs2317676 also effect on the ADP-induced platelet aggregation in IS patients taking clopidogrel [5]. Our current study demonstrated that the platelet aggregation whether AA- induced or ADP-induced was significantly higher in patients carrying high-risk interactive genotypes than in patients without carrying high-risk interactive genotypes on admission. We reason that the interactions of rs17110453A>C, rs2317676A>G, and rs16863323C>T could potentially provide these individuals with higher platelet aggregation than those without this particular three-gene-variant interaction, thereby increasing the risk for IS. However, further studies are needed to validate our findings.

Our current study have several potential limitations. First, the results of this study may have possible bias due to the relative small sample size, the two-center design of this study. Although the FPRP was calculated to evaluate the significant findings. The findings must be validated in larger, multi-center studies. Second, although we genotyped multiple known functional variants in *CYP, P2Y12*, *P2Y1* and *GPIIIa* genes, some rare functional variants may have been left undetected in this population. Finally, this study only investigated the gene–gene interactions involving fifteen variants. As previously mentioned, several gene variants have been associated with IS risk; thus, future studies involving a larger set of genetic variants must be conducted to elucidate the full extent of gene–gene interaction effects on IS pathogenesis.

## MATERIALS AND METHODS

### Study populations

This study was reviewed and approved by the Ethics Committees of the People’s Hospital of Deyang City and the Third Affiliated Hospital of Wenzhou Medical College. The study population comprised 396 IS patients and 378 healthy controls. Each of the participants provided informed consent before participating in this study. The study was registered at http://www.chictr.org/ with the unique identifier of ChiCTR-OCH-14004724.

All consecutive patients who sustained a stroke for the first time and were admitted to either of the above mentioned two hospitals were enrolled in the study. The diagnosis of IS was confirmed by brain magnetic resonance imaging. Inclusion criteria were as follows: (1) age ≥ 40 years old; (2) IS was categorized as either AT subtype or SAD subtype, according to the Trial of Org 10172 in Acute Stroke Treatment (TOAST) criteria [31]. The exclusion criteria were as follows: (1) cardiogenic cerebral embolisms or any other determined or undetermined etiology of IS; (2) family history of apoplexy or a previous history of strokes or MI; or (3) cerebral hemorrhage.

The healthy volunteers who served as controls were selected from outpatients with no history of stroke as confirmed by medical history as well as physical and laboratory examinations at our centers. They had no family history of stroke and were not genetically related to the IS patients.

All subjects had no medical or family history of hereditary diseases. Participants enrolled in the present study were free of arthritis, infection, cancer, blood disease, autoimmune diseases as well as severe heart, lung, liver, kidney, or thyroid diseases. The overall response rate was approximately 94% (396/421) for IS cases and 93% (378/406) for controls.

A detailed medical history and information on stroke risk factors was obtained from each participant, including age, gender, hypertension, diabetes mellitus, cigarette smoking, alcohol intake, TC, TG, LDL-C, and hemoglobin A1C. Diabetes mellitus was defined as when a subject had a fasting glucose level >7.8 mmol/L or >11.1 mmol/L at 2 h after an oral glucose challenge or if hypoglycemic drugs were being taken. Hypertension was defined as the mean of three independent measurements of blood pressure ≥140/90 mmHg or if antihypertensive drugs were being taken.

### Selection of CYP SNPs and genotyping

In this study, fifteen SNPs in *CYP3A4, CYP3A5, CYP2C8, CYP2C9, CYP2C19, P2Y12*, *P2Y1* and *GPIIIa* genes were selected from the NCBI database (http://www.ncbi.nlm.nih.gov/SNP), based on the following criteria:(1) SNPs that have been assessed in previous studies [4, 5, 8, 9, 14, 15], (2) SNPs leading to amino acid changes.

Genotyping was performed on genomic DNA extracted from periphery blood using the matrix-assisted laser desorption/ionization time of flight mass spectrometry method according to our previous studies [4, 5]. Genotype call was performed in real-time with MassARRAY RT software version 3.0.0.4 and analyzed using a MassARRAY Typer software version 3.4 (Sequenom Inc., San Diego, CA).

Each allele of these SNPs was classified by its known effect on function [4, 5]. For each gene, subjects were dichotomized *a priori* into two groups based on whether or not they possessed at least one mutant allele.

### Platelet aggregation tests

Venous blood (3 mL) was drawn from an antecubital vein on admission. Platelet aggregation was measured by light transmittance aggregometry (LTA). The procedures and consistency tests were performed as described in our previous studies [4, 5, 32]. Platelet aggregation was recorded as changes in light transmission. The results of optical platelet aggregometry are presented as the amplitude of light transmittance at five minutes after addition of the agonist 0.5 mM AA and 10 μM ADP with a BioData PAPS-4 platelet aggregometer (Helena Laboratories, Beaumont, TX, USA).

### Statistical analysis

Based on a suggested sample size requirement for detecting gene-gene interactions [33], we expected that our sample size of 360 patients and 360 controls would sufficiently provide 80% power at the 5% significance level calculated using three genetic models: the additive model, the dominant model, and the recessive model.

All statistical analyses were performed using SPSS 16.0 (SPSS Inc., Chicago, IL). The χ^2^ test was used to analyze the deviation from Hardy–Weinberg equilibrium for genotype frequencies and to compare genotype frequencies. Continuous variables were compared between patients with IS and controls using the Student’s t-test. Discrete variables were compared using the χ^2^ test; or when expected cell frequencies were small, Fisher’s exact tests were conducted.

For gene-gene interaction analyses, the GMDR method was applied (Beta, version0.7, www.healthsystem.virginia.edu/internet/addiction-genomics/Software) [9, 17]. The GMDR computed the maximum likelihood estimates and the scores of all individuals under the null hypothesis. The *P* value was determined by using the sign test, a robust nonparametric test implemented in the GMDR software. Permutation test was applied for multiple testing corrections. The statistical significance was determined by comparing the average prediction error from the observed data with the distribution of average prediction errors. Permutation test (combined with cross-validation) can minimize false-positive results due to multiple tests. This model with the minimum prediction error, the maximum cross-validation consistency score, and a *P* value of 0.05 or less (derived automatically from the sign test in the GMDR software) was considered as the best model. Furthermore, multivariate logistic regression analysis was performed to adjust covariate risk factors to assess the independent contribution of gene-gene interactions on IS risk. The relative risk of a genotype and the prevalence of IS were expressed with odds ratios (ORs) and 95% confidence intervals (CIs).

The FPRP was calculated to evaluate the significant findings [34]. We set 0.2 as an FPRP threshold and assigned a prior probability of 0.1 to detect an odds ratio (OR) of 0.67/1.50 (protective/risk effects) for an association with genotypes. Only the significant result with an FPRP value less than 0.2 was considered a noteworthy finding.

## CONCLUSION

In present study, single-gene variant analysis showed no significant differences in the genotype distributions of the fifteen variants in *CYP, P2Y12*, *P2Y1* and *GPIIIa* genes between IS patients and controls. However, the GMDR analysis showed a significant gene-gene interaction among rs17110453A>C, rs2317676A>G, and rs16863323C>T; and this gene-gene interaction may increase susceptibility to IS in Chinese populations. The combinational analysis used in this study may provide further insight into the complex pathogenesis of IS.

## DECLARATIONS

### Ethics approval and consent to participate

The study protocol was approved by the Ethics Committee of the People’s Hospital of Deyang City and the Third Affiliated Hospital of Wenzhou Medical University. Written informed consent was obtained from each patient prior to study enrollment.

### Consent for publication

Consent for publication is obtained from all participants.

## Abbreviations

CYP: cytochrome P450
P2Y: platelet membrane receptor
GPIIIa: glycoprotein IIIa
IS: ischemic stroke
AA: arachidonic acid
EETs: epoxyeicosatrienoic acids
SNPs: single nucleotide polymorphisms
AT: atherothrombotic
SAD: small artery disease
TOAST: Trial of ORG 10172 in the Acute Stroke Treatment
MI: myocardial infarction
DM: diabetes mellitus
TC: total cholesterol
LDL-C: low-density lipoprotein cholesterol
HDL-C: high-density lipoprotein cholesterol
LTA: light transmittance aggregometry
ADP: adenosine diphosphate
OR: odds ratio
CI: confidence interval
FPRP: false-positive report probability
